# Rab18 Dynamics in Adipocytes in Relation to Lipogenesis, Lipolysis and Obesity

**DOI:** 10.1371/journal.pone.0022931

**Published:** 2011-07-28

**Authors:** Marina R. Pulido, Alberto Diaz-Ruiz, Yolanda Jiménez-Gómez, Socorro Garcia-Navarro, Francisco Gracia-Navarro, Francisco Tinahones, José López-Miranda, Gema Frühbeck, Rafael Vázquez-Martínez, Maria M. Malagón

**Affiliations:** 1 Department of Cell Biology, Physiology and Immunology, University of Córdoba, Córdoba, Spain; 2 Centro de Investigación Biomédica en Red Fisiopatología de la Obesidad y Nutrición, Córdoba, Spain; 3 Instituto Maimónides de Investigación Biomédica (IMIBIC), Córdoba, Spain; 4 Servicio de Endocrinología y Nutrición, Hospital Clínico Virgen de la Victoria, Málaga, Spain; 5 Lipids and Atherosclerosis Unit, Hospital Universitario Reina Sofía, Córdoba, Spain; 6 Metabolic Research Laboratory, University of Navarra, Pamplona, Spain; Cardiff University, United Kingdom

## Abstract

Lipid droplets (LDs) are organelles that coordinate lipid storage and mobilization, both processes being especially important in cells specialized in managing fat, the adipocytes. Proteomic analyses of LDs have consistently identified the small GTPase Rab18 as a component of the LD coat. However, the specific contribution of Rab18 to adipocyte function remains to be elucidated. Herein, we have analyzed Rab18 expression, intracellular localization and function in relation to the metabolic status of adipocytes. We show that Rab18 production increases during adipogenic differentiation of 3T3-L1 cells. In addition, our data show that insulin induces, via phosphatidylinositol 3-kinase (PI3K), the recruitment of Rab18 to the surface of LDs. Furthermore, Rab18 overexpression increased basal lipogenesis and Rab18 silencing impaired the lipogenic response to insulin, thereby suggesting that this GTPase promotes fat accumulation in adipocytes. On the other hand, studies of the β-adrenergic receptor agonist isoproterenol confirmed and extended previous evidence for the participation of Rab18 in lipolysis. Together, our data support the view that Rab18 is a common mediator of lipolysis and lipogenesis and suggests that the endoplasmic reticulum (ER) is the link that enables Rab18 action on these two processes. Finally, we describe, for the first time, the presence of Rab18 in human adipose tissue, wherein the expression of this GTPase exhibits sex- and depot-specific differences and is correlated to obesity. Taken together, these findings indicate that Rab18 is involved in insulin-mediated lipogenesis, as well as in β-adrenergic-induced lipolysis, likely facilitating interaction of LDs with ER membranes and the exchange of lipids between these compartments. A role for Rab18 in the regulation of adipocyte biology under both normal and pathological conditions is proposed.

## Introduction

White adipose tissue is essential for the maintenance of energy homeostasis, in terms of its role both as an endocrine organ and as the main energy reservoir of the body, responsible for storing energy in the form of triglycerides (TAG) during periods of energy excess and releasing it as free fatty acids (FFAs) to be used as an energy source by other tissues during times of energy deprivation. TAG accumulation (*i.e.*, lipogenesis) and hydrolysis (*i.e.*, lipolysis) in adipocytes are primarily controlled by insulin and catecholamines [1], [2], [3], which, together with other endocrine and paracrine/autocrine factors, ensure correct lipid storage and utilization [4], [5], [6]. Alterations in adipocyte lipid metabolism, as occur in obesity and lipodystrophy, are associated with insulin resistance, which represents a major risk factor for the development of type 2 diabetes, hepatic steatosis, hypertension and cardiovascular disease [7], [8].

Intracellularly, adjustments in lipid metabolism take place in specialized organelles, the lipid droplets (LDs), in which TAG and other neutral lipids accumulate in a central core that is surrounded by a phospholipid monolayer and a coat of associated proteins [9], [10]. Specifically, besides the enzymes involved in lipid biosynthesis [diacylglycerol acyltransferase 2 (DGAT2)] [11] and hydrolysis [hormone-sensitive lipase (HSL), adipose triglyceride lipase (ATGL)] [12], LDs are decorated with a range of proteins that also contribute to the control of lipid storage and mobilization and regulate LD biogenesis and movement [13]. Among these, PAT (perilipin-adipophilin-Tip47) proteins [14] have been extensively studied in terms of their association with the LD surface and their role in controlling LD function [reviewed by 15]. In particular, perilipin has been shown to have a dual action on LDs promoting TAG storage under basal conditions while facilitating TAG lipolysis in response to β-adrenergic stimulation [15]. In addition to the PAT family, recent proteomic studies carried out on purified LDs have led to the identification of a large number of other LD-associated proteins that belong to the membrane traffic (*e.g.*, Rab proteins) and fusion machineries (*e.g.*, SNARE proteins) [13], [16], [17], which highlights the dynamic nature of these organelles. Therefore, it is now clear that LDs act as integration centers of lipid metabolism in adipocytes and understanding the intracellular mechanisms that control LD biology holds the key to building a unified picture of the (patho)physiological function of adipocytes.

Recently, the small GTPase Rab18 has been identified as a novel protein associated with the LD surface in 3T3-L1 adipocytes as well as in other LD-containing non-adipocyte cell lines [13], [16], [18], [19], [20]. This GTPase has been found to regulate intracellular membrane trafficking events in various different cells types [21], [22], [23]. In adipocytes, the specific localization of Rab18 around LDs together with the observations that induction of lipolysis in 3T3-L1 cells increased Rab18 recruitment to the surface of LDs [18] and that Rab18 overexpression caused close membrane apposition between LDs and ER-derived membranes [20], led to the proposal that this GTPase might be involved in mobilizing lipid esters stored in LDs. However, the exact contribution of Rab18 to lipid metabolism and the mechanisms regulating Rab18 function in adipose tissue remain to be elucidated. In the present study, we show that, in addition to mediating β-adrenergic action in adipocytes, Rab18 is a downstream effector of the metabolic changes induced by insulin in this cell type. Our data provide novel experimental evidence supporting the involvement of this GTPase as a key mediator in the bidirectional trafficking of lipids between LDs and the ER. Finally, we explore the regulation of Rab18 expression in human adipose tissue as a function of sex, adipose tissue localization and obesity.

## Results

### Rab18 expression during differentiation of 3T3-L1 cells to adipocytes

**Figure 1:**
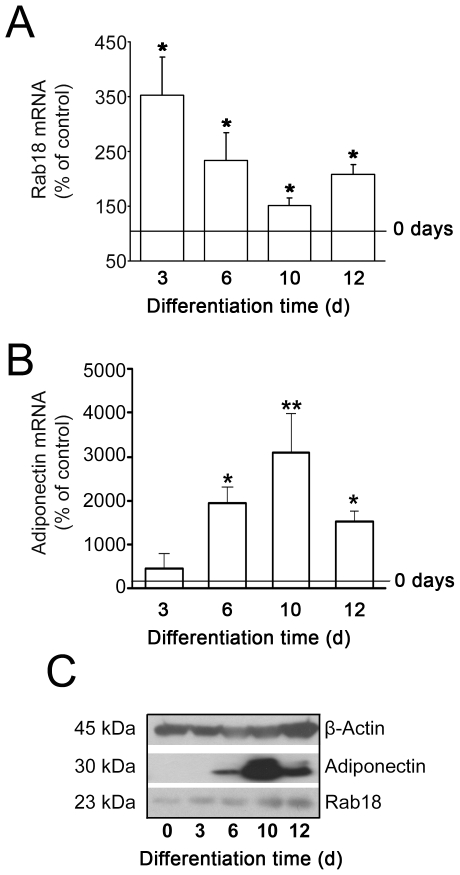
Rab18 expression during differentiation of 3T3-L1 cells into adipocytes. (A) Quantitative RT-PCR analysis of Rab18 mRNA levels in 3T3-L1 cells exposed to a hormonal differentiation cocktail for 0, 3, 6, 10 and 12 days. Gene expression was expressed as the ratio of target gene concentration to the concentration of a housekeeping gene, the 18S rRNA (2.0×10^8^±7.7×10^7^ and 3.2×10^13^±2.2×10^12^ Rab18 and 18S cDNA copies, respectively, per 0.1 µg total RNA in non-differentiated cells). Three independent experiments ± SEM were tested for significance using the Newman-Keul's comparison test. *, *P*<0.05 *vs.* non-differentiated cells. (B) To monitor adipogenesis, adiponectin mRNA transcripts (1.6×10^8^±1.3×10^8^ cDNA copies/0.1 µg total RNA in non-differentiated cells) were also quantified. (C) Representative immunoblots from 3 independent analyses of Rab18 and adiponectin protein content in 3T3-L1 cell extracts during differentiation. β-actin immunosignal was used as reference for protein charge.

To analyze the expression pattern of Rab18 during the differentiation of 3T3-L1 cells into adipocytes, we quantified Rab18 mRNA content at 0, 3, 6, 10 and 12 days of differentiation by quantitative RT-PCR. As shown in Fig. 1A, Rab18 expression was significantly higher at all time points analyzed than in non-differentiated cells, reaching a peak on day 3. At 6, 10 and 12 days of differentiation, Rab18 mRNA levels tended to decrease, although they remained approximately 75, 50 and 70% higher than in non-differentiated cells. In turn, Rab18 protein content was very low in non-differentiated cells, but it notably increased after 3 days of differentiation and remained elevated thereafter (Fig. 1C). To monitor adipogenesis, adiponectin gene expression and protein content were quantified (Figs. 1B and 1C).

### Regulation of Rab18 gene expression, protein content and subcellular localization in 3T3-L1 adipocytes

**Figure 2:**
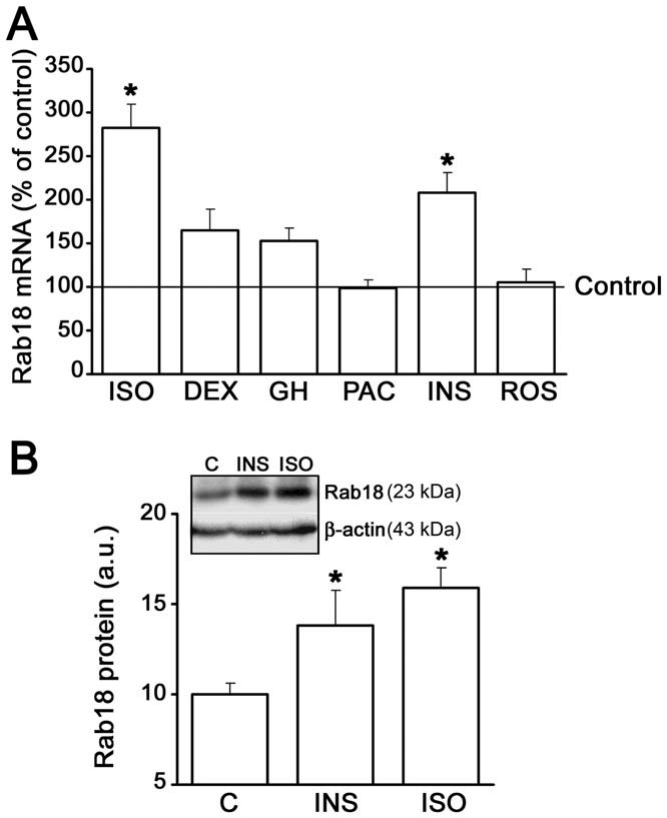
Regulation of Rab18 gene expression and protein content in 3T3-L1 adipocytes. (A) 3T3-L1 adipocytes were treated with 10 µM isoproterenol (ISO), 100 nM dexamethasone (DEX), 10 nM GH, 4.8 nM PACAP (PAC), 100 nM insulin (INS), or 10 µM rosiglitazone (ROS) for 24 h, and Rab18 mRNA levels were evaluated by quantitative RT-PCR. 18S rRNA expression was used as internal control (1.7×10^9^±9.2×10^8^ and 1.1×10^12^±1.5×10^11^ Rab18 and 18S cDNA copies, respectively, per 0.1 µg total RNA in untreated cells). Data represent the average ± SEM of 3 independent experiments. *, *P*<0.05 *vs.* untreated cells. . (B) Quantification of Rab18 band intensity in extracts from 3T3-L1 adipocytes cultured in the presence or absence of 100 nM insulin (INS) or 10 µM isoproterenol (ISO) for 24 h s. β-actin immunostaining was used as loading control. Data are expressed as the mean ± SEM of 3 independent experiments. *, *P*<0.05 *vs.* untreated cells. A representative immunoblot of Rab18 and β-actin is shown.

In order to ascertain the specific contribution of Rab18 to adipocyte function, we first studied how different extracellular stimuli known to control lipid metabolism affect Rab18 production and its subcellular localization in 3T3-L1 adipocytes. Regarding Rab18 expression, 24-h treatments with either 100 nM insulin or 10 µM isoproterenol significantly increased Rab18 mRNA levels, which accounted for by 183% and 108% above baseline levels, respectively (Fig. 2A). Other treatments, such as 100 nM dexamethasone and 10 nM GH, also tended to increase Rab18 gene expression, although these effects were not statistically significant. Finally, exposure of 3T3-L1 adipocytes to 4.8 nM pituitary adenylate-cyclase activating polipeptide-38 (PACAP38) or 10 mM rosiglitazone did not alter Rab18 transcript levels. In view of these results, we chose the strongest inductors of Rab18 expression (namely, insulin and isoproterenol) to explore their effects on Rab18 protein content. As depicted in Fig. 2B, 24-h treatment with either 100 nM insulin or 10 µM isoproterenol elicited increases in Rab18 protein content of 38% and 59% as compared to non-stimulated conditions, respectively. These data indicate that Rab18 production is regulated by specific regulatory inputs reaching the adipocytes and suggest that the GTPase may form part of the intracellular machinery activated by such factors.

**Figure 3:**
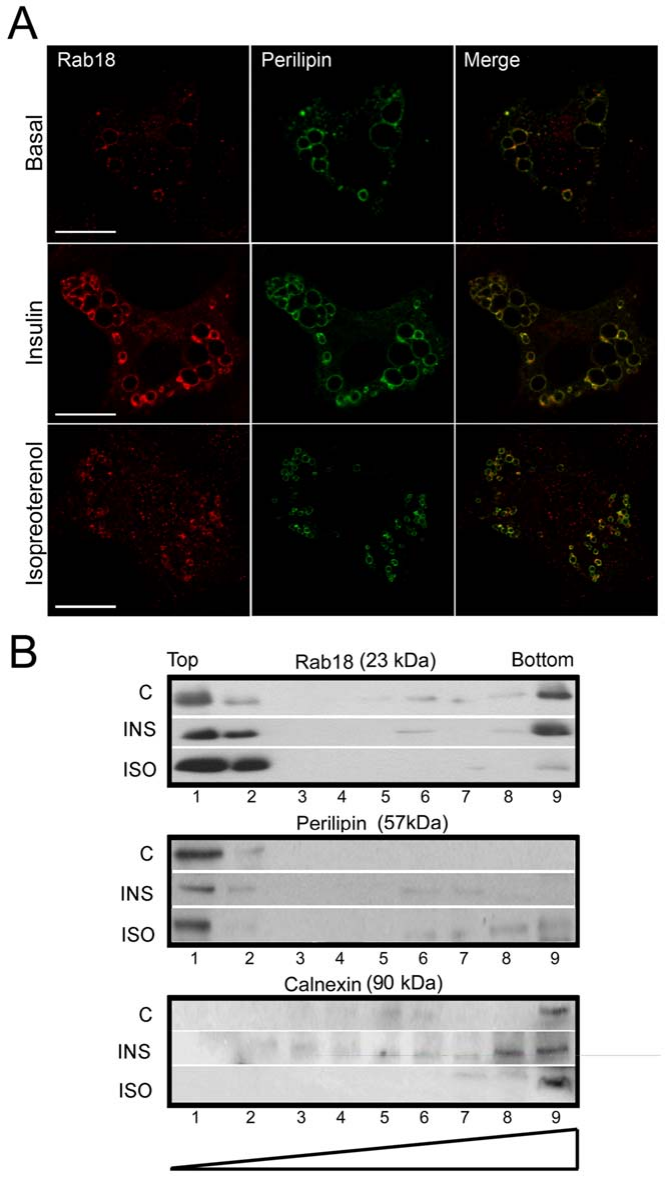
Rab18 subcellular localization in 3T3-L1 adipocytes under insulin or isoproterenol stimulation. (A) Confocal microscope images of 3T3-L1 cells under basal conditions (top panels) or challenged with 100 nM insulin (middle panels) or 10 µM isoproterenol (bottom panels) for 4 h and co-immunostained for Rab18 (red) and the LD-associated protein perilipin (green). Colocalization of the two immunosignals can be seen in the most right panels (yellow). Scale bars, 5 µm. (B) Immunoblots of sucrose gradients from 3T3-L1 cells under non-stimulated conditions and after 4-h treatments with 100 nM insulin and 10 µM isoproterenol. Perilipin and calnexin immunoreactivities were used as markers of LD-enriched fractions (light fractions) and microsomal fractions (heavy fractions), respectively. One representative experiment out of three with similar results is shown.

Previously, several studies have reported that Rab18 localizes at the surface of LDs [18], [20] and that isoproterenol treatment increases this association [18]. Inasmuch as in the present work we have found that, like isoproterenol, insulin modulates Rab18 production in adipocytes, we asked whether this hormone could also affect intracellular localization of Rab18. To this end, 3T3-L1 adipocytes were subjected to a 4-h treatment with 100 nM insulin and co-immunostained with antibodies against Rab18 and the LD-surface resident, non-exchangeable protein perilipin [24], and visualized under a confocal microscope. As a positive control, cells were treated with 10 µM isoproterenol and processed for immunocytochemistry. Figure 3A shows representative images of cells under basal conditions and cells treated with either insulin or isoproterenol. As shown, in non-treated cells Rab18 a certain level of immunoreactivity was detected around the LDs, displaying a significant degree of overlap with perilipin immunofluorescence (PC = 0.19±0.05; n = 8 cells; Fig. 3A, top panels). Insulin administration notably enhanced Rab18 immunoreactivity around LDs and, hence, colocalization with perilipin (PC = 0.38±0.07; n = 9 cells; Fig. 3A, mid panels). Likewise, colocalization of Rab18 and perilipin also significantly increased in response to the β-adrenergic agonist (PC = 0.34±0.04; n = 8 cells; Fig. 3A, bottom panels). To confirm the effectiveness of the treatments, we measured the average LD size under each condition. It was found that LDs were significantly larger in insulin-treated cells than in controls, which is consistent with an increase in lipid deposition, while LDs were smaller after isoproterenol stimulation, likely due to increased TAG lipolysis (1.39±0.13, 1.82±0.10, and 0.18±0.02 µm^2^ in control, insulin- and isoproterenol-treated cells, respectively; *P*<0.05).

The effect of insulin and isoproterenol on Rab18 association with LDs was also explored using subcellular fractionation in a sucrose density gradient followed by immunoblotting of the resulting fractions. Under basal conditions, the Rab18 immunosignal was mainly localized in the perilipin-enriched and microsomal fractions (top and bottom of the gradient respectively), although a degree of immunoreactivity was also noticeable in some cytosolic fractions (Fig. 3B). After insulin treatment, Rab18 immunolabeling was 43% higher in the perilipin-enriched fractions, whereas the amount of protein present in the microsomal fraction remained the same as in control conditions. In addition, insulin provoked a slight decrease in Rab18 immunoreactivity in cytosolic fractions. Regarding isoproterenol, this treatment induced a 48% increase in Rab18 immunoreactivity associated with perilipin-enriched fractions, similar to that previously reported [18], and a concomitant decrease in the microsomal and cytosolic fractions. In sum, these findings indicate that, as in isoproterenol-induced β-adrenergic receptor activation, the intracellular signaling pathway initiated by insulin involves Rab18 recruitment to the surface of LDs, which further supports the notion of a role for this GTPase in the metabolic response of 3T3-L1 adipocytes to this hormone.

**Figure 4:**
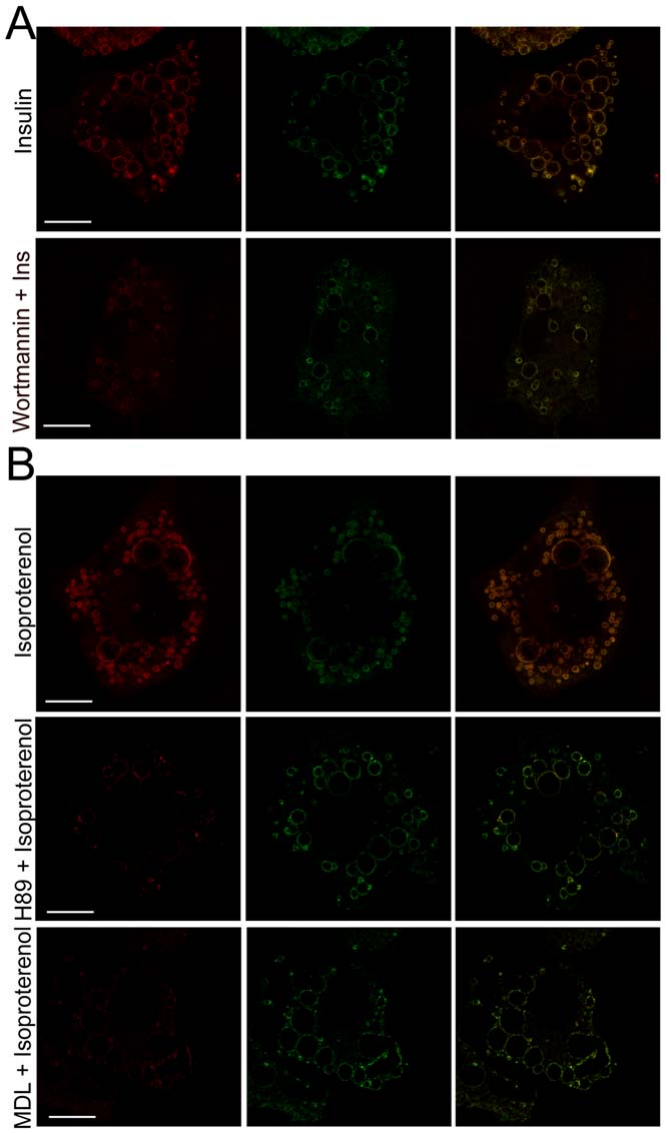
Intracellular signaling pathways mediating the effect of insulin and isoproterenol on Rab18 association with LDs. (A) Representative confocal images of 3T3-L1 adipocytes treated with 100 nM insulin for 4 h in the absence (top panels) or presence of the PI3K blocker wortmannin (1 µM). (B) Representative confocal images of 3T3-L1 adipocytes treated with 10 µM isoproterenol for 4 h in the absence (top panels) or presence of the PKA blocker H89 (1 µM; middle panels) or the adenylate cyclase blocker MDL 12,330 (1 µM; bottom panels). After treatment, cells were co-immunostained for Rab18 (red) and perilipin (green). Colocalization of the two proteins is shown in the images on the far right (yellow). Scale bars, 5 µm.

We next examined the intracellular signaling pathways that mediate the effect of insulin and isoproterenol in Rab18 association to LDs. In adipocytes, most of the metabolic actions of insulin are mediated by activation of phosphatidilinositol-3-kinase (PI3K) that phosphorylates Akt, the latter being responsible for the activation of various different substrates [25], [26]. We therefore investigated whether the increase in Rab18 at the surface of LDs induced by insulin is a consequence of activation of PI3K/Akt by quantifying the degree of colocalization between Rab18 and perilipin immunolabeling in cells treated with insulin in the presence of the PI3K blocker wortmannin. This showed that in the presence of wortmannin, insulin was prevented from increasing colocalization of Rab18 and perilipin at the surface of LDs (PC = 0.50±0.09 *vs.* 0.36±0.02 in insulin-treated cells in the absence and presence of wortmannin, respectively; *P*<0.05; Fig. 4A). These data indicate that insulin exerts its effect on the intracellular localization of Rab18 *via* activation of the PI3K/Akt signaling cascade. In the case of isoproterenol, its effect on lipolysis in adipocytes is mediated by activation of β-adrenergic receptors, which initiates the adenylate cyclase (AC)/cAMP/protein kinase A (PKA) pathway [27]. PKA phosphorylates hormone sensitive lipase (HSL), which then translocates to the LD surface and triggers the enzymatic reactions that lead to fatty acid hydrolysis [28]. In the current work, we demonstrate that the isoproterenol-induced effect on Rab18 localization is mediated by activation of the AC/cAMP/PKA pathway, inasmuch as blockade of either AC by treating cells with MDL 12,330A or PKA by using H89 prior to isoproterenol administration significantly decreased colocalization of Rab18 and perilipin immunosignals on the LD surface (PC = 0.44±0.05 *vs.* 0.27±0.03 and 0.26±0.07 in isoproterenol-treated cells in the absence and presence of MDL 12,330A or H89, respectively; *P*<0.05; Fig. 4B), reaching levels comparable to those obtained in non-treated cells.

### Rab18 and LD apposition to ER membranes

**Figure 5:**
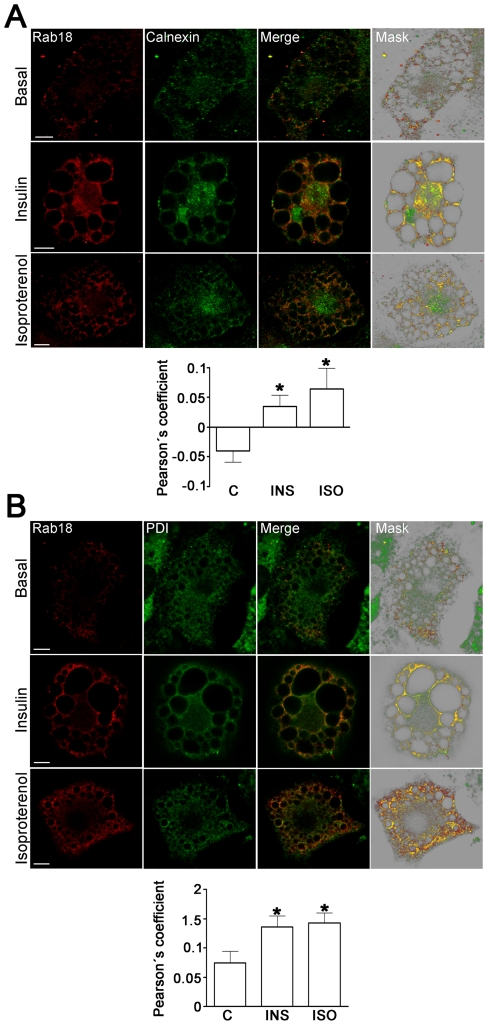
Effects of insulin and isoproterenol on Rab18 localization in relation to the ER. Representative confocal images of 3T3-L1 adipocytes under basal conditions or treated with 100 nM insulin or 10 µM isoproterenol for 4 h. After treatment, cells were co-immunostained for Rab18 (red) and the ER markers calnexin (A) or protein disulfure isomerase (B). In all cases, regions of interest containing a group of Rab18-positive LDs are presented. The colocalization channel was isolated using Imaris 6.4 (Bitplane) and shown alone in the images on the far right. Scale bars, 2 µm. To quantify the degree of colocalization, Pearson' coefficients were calculated for each experimental condition and represented as the mean ± SEM of, at least, 10 cells per experimental group. *, *P*<0.05 *vs.* untreated cells.

In a previous study, Ozeki and coworkers [20] found that overexpression of exogenous Rab18 induced apposition of LD and ER membrane surfaces. Herein, we have extended these findings concerning endogenous Rab18 by demonstrating that rapprochement of Rab18-bearing LDs to ER membranes is a process regulated by extracellular inputs reaching the adipocytes, including both insulin and β-adrenergic stimulation. Specifically, colocalization analysis of Rab18 and the ER markers calnexin (Fig. 5A) and protein disulfide isomerase (PDI; Fig. 5B) revealed that, under basal conditions, there is little colocalization between this GTPase surrounding LDs and the ER. Accordingly, the degree of colocalization between Rab18 and calnexin or PDI immunoreactivities was found to be very low in non-stimulated cells. On the other hand, after insulin or isoproterenol administration, colocalization between Rab18 and both ER markers notably increased.

### Rab18 contribution to lipogenesis

To investigate the specific contribution of Rab18 to LD physiology, we carried out Rab18 overexpression and silencing studies in 3T3-L1 cells. For overexpression experiments, cells were transiently transfected with a vector coding for either Rab18 or the constitutive active mutant Rab18(Q67L) fused to GFP. In all cases, transfection efficiency was over 90% as assessed by fluorescence microcopy (data not shown). Supplementary Fig. S1 (panel A) shows the intracellular distribution of GFP-Rab18 in 3T3-L1 adipocytes under basal conditions and after insulin treatment. As shown, exogenously expressed Rab18 mainly localized around a particular population of Oil-Red O labeled LDs, similar to the pattern found for endogenous Rab18. The specific GFP-Rab18 distribution around LDs can be seen in more detail in the rendered image (Fig. S1, panel B), which illustrates Oil-Red O stained LDs, one showing a ring-shaped coating of GFP-Rab18. Similar to what had been previously observed in non-transfected cells, insulin increased mean LD size in mock-transfected 3T3-L1 cells, an effect that was not observed when cells were challenged with isoproterenol (Fig. S1, panel C). Specifically, comparison of the average LD size from GFP-expressing cells versus GFP-Rab18-expressing cells revealed that, in the latter group, LDs were significantly larger than in mock-transfected cells both under basal conditions and after insulin administration or isoproterenol treatment (Fig. S1, panel C). The strong effect of insulin on LD size in Rab18-overexpressing cells as compared to GFP-transfected cells is noteworthy, and suggests that Rab18 may facilitate insulin-mediated lipid uploading into LDs.

**Figure 6:**
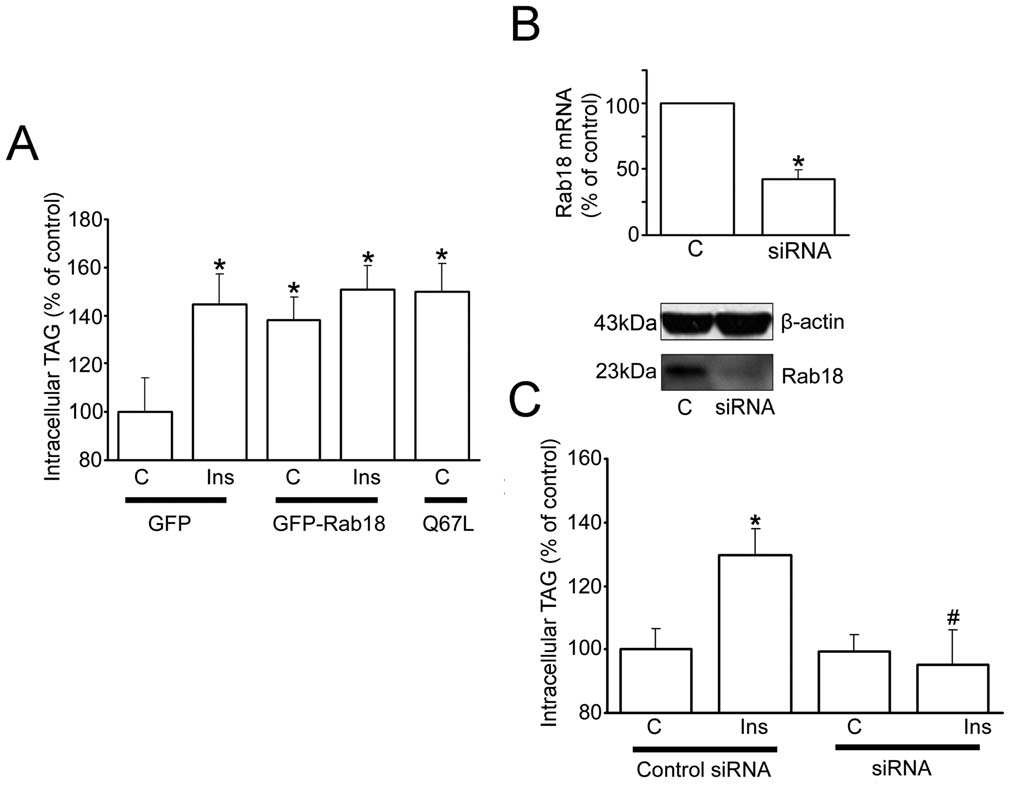
Effect of Rab18 overexpression and silencing on insulin-induced lipogenesis. (A) The effect of Rab18 overexpression on basal and insulin-induced lipogenic activity in 3T3-L1 adipocytes was assessed by transfecting cells with GFP-tagged versions of wild-type Rab18 or the constitutively active mutant Rab18(Q67L) for 48–72 h. In parallel, a group of cells (controls) were transfected a vector expressing GFP alone. GFP fluorescence served to confirm transfection efficiencies higher than 90% for each experiment prior to further experimental manipulation. After 4-h treatment, cells were lysed and intracellular TAG content was quantified. TAG content was normalized to total protein content and expressed as the mean ± SEM of 6 independent experiments. (B) Rab18 silencing was achieved by transfecting 3T3-L1 cells with a mouse Rab18 specific siRNA. As control, a group of cells were transfected with a scramble negative control siRNA. Rab18 knock down was confirmed by quantitative RT-PCR and Western blot. (C) After 72 h, cells were challenged to 100 nM insulin for 4 h, harvested and lysed. TAG was content quantified as indicated above and expressed as the mean ± SEM of 6 independent experiments. *, *P*<0.05 and **, *P*<0.01 *vs.* untreated control cells. #, *P*<0.05 *vs.* insulin-treated control cells.

Quantification of the lipogenic activity in Rab18-expressing 3T3-L1 cells revealed that, under basal conditions, Rab18 overexpression provoked a 38% increase in triglyceride content, and this seemed to be a maximal level, as insulin treatment was unable to induce further increases. The stimulatory effect of Rab18 on basal lipogenesis was reproduced by transfection of cells with the constitutively active mutant Rab18(Q67L) (Fig. 6A).

**Figure 7:**
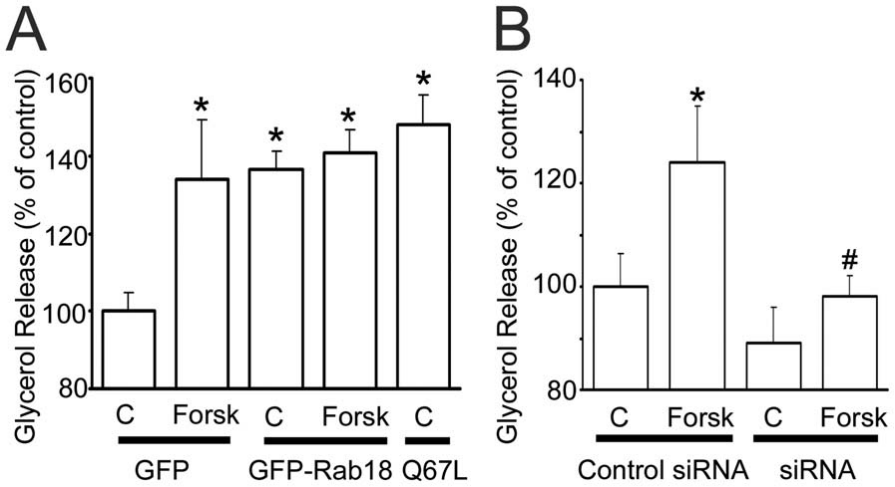
Effect of Rab18 overexpression and silencing on forskolin-induced lipolysis. (A) The effect of Rab18 overexpression on basal and forskolin-induced lipolysis was evaluated by transfecting 3T3-L1 adipocytes with GFP-tagged, wild-type Rab18 or the constitutively active mutant Rab18(Q67L) for 48–72 h. In each experiment, a group of cells (controls) were transfected a vector expressing GFP alone. GFP fluorescence served to confirm transfection efficiencies higher than 90% for each experiment prior to further experimental manipulation. After 30 min in the presence or absence of forskolin, the culture medium was collected and glycerol release measured with Free Glycerol Reagent kit (Sigma-Aldrich). Glycerol concentration in the medium was normalized to total protein and expressed as the mean ± SEM of 5 independent experiments. (B) Rab18 silencing was achieved by transfecting 3T3-L1 cells with a mouse Rab18 specific siRNA. After 72 h, cells were challenged with 50 µM forskolin for 30 min. Glycerol release was quantified as indicated above and expressed as the mean ± SEM of 5 independent experiments. *, *P*<0.05 *vs.* untreated control cells. #, *P*<0.05 *vs.* forskolin-treated control cells.

For silencing studies, 3T3-L1 cells were transfected with a synthetic siRNA, which reduced Rab18 mRNA levels by 60%, as evaluated by real-time RT-PCR, and lowered endogenous levels of Rab18 protein by 70%, as assessed by immunoblotting (Fig. 6B). As shown in Fig. 7C, reduction in Rab18 expression did not modify basal lipogenic activity but abrogated insulin-stimulated lipogenesis. Overall, results from our overexpression and silencing studies suggest that Rab18 facilitates triglyceride accumulation in LDs.

### Rab18 contribution to lipolysis

Given the increased association of Rab18 with the surface of LDs upon stimulation of lipolysis ([18] and our present data), we assessed how Rab18 overexpression and knock-down affect the lipolytic activity of adipocytes. Specifically, we found that GFP-Rab18 overexpression induced a 44% increase in glycerol release under basal conditions, mimicking the effect of forskolin in mock-transfected cells (Fig. 7A). Moreover, forskolin did not cause any further enhancement in the lipolytic activity of cells. Likewise, Rab18 constitutive active mutant Rab18(Q67L) also significantly increased basal lipolysis to the same extent as seen with the wild-type GTPase (Fig. 7A). In order to ascertain whether this effect was restricted to fully differentiated cells, we overexpressed GFP-Rab18 at an early stage of the differentiation process (4 days), when cells already present β-adrenergic receptors [29] and adenylate-cyclase activity is enhanced in response to isoproterenol [30], and evaluated the effect of forskolin on lipolysis two days after transfection. These results revealed that Rab18 overexpression exerted the same effect on the lipolytic rate in cells differentiated for 6 days (data not shown) as those differentiated for 10 days.

Regarding the effect of Rab18 silencing on lipolysis, glycerol release was unaltered in untreated cells while forskolin administration failed to stimulate lipolysis, as compared to cells transfected with the negative control siRNA (Fig. 7B). These results provide strong evidence for a role for Rab18 in TAG hydrolysis and, together with the data obtained on insulin, suggest that this GTPase is a molecular player common to the lipolytic and lipogenic pathways converging in LDs.

### Rab18 expression in human adipose tissue

We assessed the expression of Rab18 in both subcutaneous and omental adipose tissue samples from lean (BMI = 21.8±1.3 kg/m^2^; n = 8 men and 4 women) and obese (BMI = 48.2±2.6 kg/m^2^; n = 16 men and 18 women) individuals. This latter group was divided into normoglycemic (NG; n = 5 men and 6 women), insulin resistant (IGT; n = 5 men and 6 women), and type 2 diabetic (T2D; n = 6 men and 6 women) subjects. We first explored the subcellular distribution of the GTPase in isolated mature adipocytes from lean individuals, finding that Rab18 immunoreactivity is associated with the surface of LDs in adipocytes from both omental and subcutaneous fat (Fig. S2). Consistently, RT-PCR analysis revealed that Rab18 mRNA is expressed in both adipose tissue depots, but transcript levels of the GTPase were higher in subcutaneous adipose tissue than in omental fat irrespective of the sex of the individual (Table S1). On the other hand, Rab18 gene expression levels were higher in both fat depots in lean women with respect to lean men, although differences between the sexes only reached statistical significance in omental adipose tissue (Table S1).

**Figure 8:**
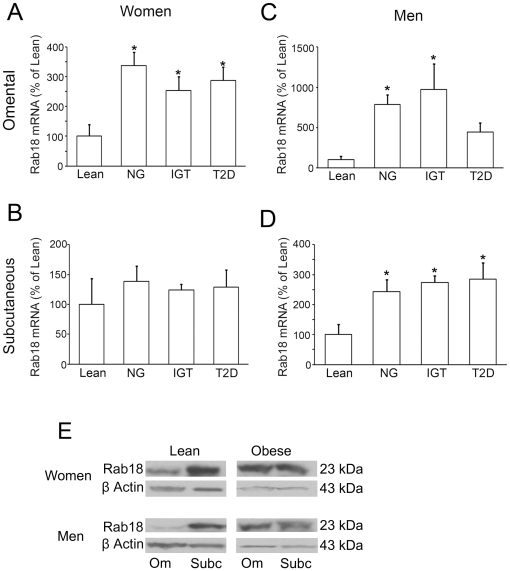
Assessment of Rab18 expression levels in human adipose tissue in relation to obesity and insulin resistance. Omental and subcutaneous adipose tissue samples were obtained from the abdominal region of women (A and B) and men (C and D) with different degrees of obesity and/or insulin-resistance: lean, obese normoglycemic (NG), obese with impaired glucose tolerance (IGT) and obese type 2 diabetic (T2D) patients. After removal, tissue samples were processed for total RNA extraction. Rab18 expression was evaluated by quantitative RT-PCR using specific primers for human Rab18. The expression of human 18S rRNA in each sample was evaluated as an internal housekeeping gene. Absolute cDNA copy number for lean patients were 2.1×10^5^±1.7×10^5^ Rab18 copies and 6.6×10^10^±5.5×10^10^ 18S copies in omental fat from women, 5.9×10^4^±3.1×10^4^ Rab18 copies and 5.7×10^10^±1.5×10^10^ 18S copies in omental fat from men, 4.9×10^4^±3.2×10^4^ Rab18 copies and 1.1×10^10^±6.9×10^9^ 18S copies in subcutaneous fat from women, and 5.2×10^4^±2.0×10^4^ Rab18 copies and 2.8×10^10^±1.3×10^9^ 18S copies in subcutaneous fat from men. *, *P*<0.05 *vs.* lean subjects. (E) Rab18 protein content in omental and subcutaneous adipose tissue samples from lean and obese women and men. In each blot, the β-actin immunosignal from the same sample was used as internal control.

When the mRNA data from the three groups of obese individuals were analyzed together, no differences for Rab18 levels were observed as a function of sex (Table S1). Similar Rab18 mRNA levels were observed in the two fat depots in obese women whereas in obese men Rab18 transcript levels were higher in subcutaneous than in omental fat (Table S1). Specifically, Rab18 expression in omental adipose tissue was upregulated by 237, 154 and 188% in normoglycemic, insulin resistant and T2D obese women, respectively, whereas no significant differences were found in the subcutaneous depot between lean and obese women (Figs. 8A and 8B). In contrast, Rab18 mRNA levels were higher in both omental and subcutaneous fat in obese men than in lean men (Fig. 8C and 8D), although the greatest differences in Rab18 mRNA content between obese and lean patients were found in the former depot. Further, Rab18 transcript content in omental adipose tissue from T2D men, though numerically higher than that observed in lean men, was not significantly different from either this group or the levels observed in the other groups of obese patients (Fig. 8C).

Finally, the protein expression of Rab18 in adipose tissue samples from lean and obese individuals exhibited a similar pattern to that observed in the gene expression analysis (Fig. 8E).

## Discussion

LDs are highly dynamic and complex organelles specialized in intermembrane lipid transport and responsible for lipid metabolism [16]. Several independent proteomic studies aimed at isolating and characterizing the protein composition of the LD surface have strongly supported this view, as they led to the identification of a plethora of proteins, many of which are directly related to intracellular membrane trafficking and fusion events (*i.e.*, Rab, SNARE and motor proteins) [17], [31], [32], [33]. Herein, we provide experimental evidence supporting a role for a member of the Rab family, Rab18, in adipogenesis as well as in mediating the lipogenic and lipolytic actions of insulin and isoproterenol, respectively, on 3T3-L1 adipocytes. Moreover, we have identified the intracellular pathways responsible for the Rab18 association with LDs and provided experimental evidence that this process entails rapprochement of LDs to ER membranes. Finally, we have demonstrated, for the first time, the presence of Rab18 in human adipose tissue and showed that the expression of this GTPase is correlated to obesity.

Studies in differentiating 3T3-L1 cells, the cell line most commonly used to study adipogenesis, revealed that Rab18 mRNA reached a maximal level on day 3 of differentiation, coinciding with the appearance of late differentiation markers (*i.e.* lipogenic and lipolytic enzymes, as well as several LD-coating proteins such as perilipin), which are responsible for the maintenance of the adipocyte phenotype in 3T3-L1 cells [34], [35]. In addition, Rab18 protein content progressively increased during differentiation. These data indicate that, as previously suggested for other Rab proteins (namely, Rab3A and Rab3D) [36], [37], Rab18 may play a role in the differentiation of 3T3-L1 fibroblasts to mature adipocytes. Notably, we found that insulin, a key component of the hormonal cocktail employed to induce this process in 3T3-L1 adipocytes [38], up-regulated Rab18 expression and increased Rab18 protein content in these cells. Furthermore, this hormone also triggered Rab18 association with LDs, a process that seems to be mediated by activation of the key upstream regulator of the metabolic actions induced by insulin in adipocytes, PI3K [25]. Similar to the pattern observed herein for Rab18, previous studies have reported that insulin induces other coating proteins to localize with LDs, including S3–12 [39], [40] and OXPAT [41]. Moreover, it has been shown that insulin, *via* PI3K, induces the activation and intracellular redistribution in adipocytes of another member of the Rab family, Rab4, which is involved in GLUT-4 vesicle trafficking [42]. These findings suggest that Rab proteins and, in particular Rab18, may be part of the intracellular machinery transducing the metabolic effects of insulin in this cell type. In line with this notion, the increase in Rab18 association with LDs induced by insulin concurred with the stimulation of intracellular TAG accumulation evoked by this hormone and, in addition, this later effect was inhibited in Rab18-silenced cells. These data support the view that insulin-induced recruitment to LDs may contribute to the lipogenic action of this hormone. A role for Rab18 in promoting lipogenesis is further backed by our observations of the increased lipogenic rate and LD size evoked by the overexpression of this GTPase in 3T3-L1 cells.

Intriguingly, the effects of insulin on the expression and intracellular localization of Rab18 were strikingly similar to those induced by β-adrenergic receptor activation which, as is widely known and also shown in this study, has an opposite effect to that of insulin on lipid metabolism. Moreover, Rab18 overexpression increased TAG hydrolysis at both early and late stages of 3T3-L1 cell differentiation, and Rab18 silencing blunted forskolin-stimulated lipolysis in differentiated 3T3-L1 cells. Overall, these data provide evidence for the participation of this GTPase in the regulation of lipolysis. When these results are viewed together with those obtained concerning the response to insulin, a role for Rab18 as a common mediator in lipogenic and lipolytic processes seems plausible. Although this dual contribution of Rab18 to the regulation of lipid metabolism may seem paradoxical, other proteins have been shown to affect these antagonistic processes in a similar manner. For example, ATGL and its coactivator CGI-58, besides their well-known role in TAG hydrolysis [43], also exhibit acyltransferase activity and, therefore, are able to promote lipogenesis [44], [45], [46]. However, the relative contribution of these contrasting functions remains unknown so far. In the case of Rab18, our findings that both insulin and isoproterenol increased the colocalization of this GTPase with ER markers, which is consistent with previous observations obtained in 3T3-L1 cells overexpressing Rab18 [20], suggest that the ER is the joining-point that enables Rab18 action to affect lipolysis and lipogenesis. In line with this, when lipid homeostasis demands fatty acid storage via insulin-induced lipogenesis, fatty acids are taken up by cytosolic fatty acid binding proteins and transported into the ER lumen for further processing to triglycerides [reviewed in 15], [24], [47]. Finally, lipid esters are loaded into LDs by a process that, although not yet well understood, appears to rely upon interaction of the surface of LDs with specialized regions of ER membranes [13]. On the other hand, during β-adrenergic receptor-induced lipolysis, the products derived from triglyceride hydrolysis are thought to be unloaded into the ER lumen as a result of the apposition and direct contact of the LD phospholipid monolayer and the ER membrane, which will ultimately cause depletion of LD lipid content and, eventually, LD regression [13]. Thus, given the close physical and functional interactions between LDs and ER membranes, combined with the general function of Rab GTPases in intracellular membrane trafficking [48], it is tempting to speculate that the presence of Rab18 on LDs may entail bringing LDs and ER membranes together, which would facilitate lipid loading and/or unloading from and to the ER reservoir depending on the metabolic demands of adipocytes. Consistent with this idea, a role for Rab18 in regulating tethering and/or fusion events on ER membranes has been recently proposed [21], [22], [23]. Mechanistically, Rab proteins accomplish their functions by activating downstream effectors that ultimately affect the molecular machinery of a particular process [49]. For example, Rab27A is involved in controlling trafficking of secretory granules in a wide variety of secretory cells through interaction with cell-type- and tissue-specific Rab27A effectors [50]. In particular, when Rab27A recruits Slp4-a, cells dramatically decrease their hormone secretion rate [51], whereas when Rab27A binds rabphilin, the secretory activity of the cells greatly increases [52]. In this scenario, it is plausible that, depending on the extracellular input reaching adipocytes (*i.e.* insulin or catecholamines), Rab18 interacts with different effector proteins, which in turn results in the repositioning of LDs to appropriate locations relative to specific subdomains of the ER specialized in either lipogenesis or lipolysis [13], [16]. Alternatively, given the demonstrated heterogeneity of LDs within cells in terms of their repertoire of coating proteins [12], lipogenic or lipolytic stimuli may target Rab18 to distinct LD subpopulations specialized in lipid storage or breakdown. Unfortunately, the specific Rab18 effectors in adipocytes remain to be identified. Ongoing experiments in our laboratory are focused on the identification of Rab18 interacting proteins in adipose tissue, which would pave the way for a better understanding of Rab18 function in relation to lipid metabolism.

Based on our findings on Rab18 in 3T3-L1 cells and given the relationship between human obesity and obesity-associated T2D and altered adipocyte lipid metabolism [53], we examined Rab18 in human adipose tissue in subjects with different metabolic conditions. To our knowledge, this is the first report on the characterization of a member of the Rab family in human fat in relation to sex, depot, degree of adiposity and insulin sensitivity. In particular, we have demonstrated that, similarly to what is seen in 3T3-L1 cells ([18] and this study), Rab18 also coats the surface of LDs in human adipocytes. We found both sex- and depot-related differences in Rab18 expression in non-obese subjects, with women *vs.* men and subcutaneous *vs.* omental adipose tissue showing higher mRNA and protein levels of this GTPase. These differences might reflect the distinct, sex-dependent metabolic activity of omental and subcutaneous adipose tissue [54]. Further, it has been shown that, compared with omental adipocytes, subcutaneous adipocytes in non-obese women are larger, have higher LPL activity, and are more lipolytic on an absolute basis, which may underlie the higher fat storage capacity in this depot in women [55]. Additionally, in both sexes subcutaneous adipocytes are more sensitive to insulin actions than visceral adipocytes [reviewed by 56], yet the opposite holds true for catecholamines [54], [56]. On the other hand, no differences sex-related differences in Rab18 gene expression were detectable in obese individuals, mainly due to the significant upregulation of Rab18 mRNA levels observed in both fat depots in morbidly obese men. Indeed, except for the subcutaneous depot in women, obesity was associated with an increase in Rab18 expression, which suggests that upregulation of this GTPase may be an appropriate response to managing energy excess. Nevertheless, these results were unexpected as recent studies in humans, based either on microarray data [57] and on the analysis of the expression of specific proteins involved in lipolysis (*i.e.* HSL; [58]) or lipogenesis (*i.e.* FAS; [59]), suggest that both processes are decreased in severely obese subjects. In this scenario, it is tempting to speculate that the enhanced expression of Rab18 in obese individuals is an adaptive response to overcome the alterations in lipid metabolism occurring in obesity. Finally, although we observed a tendency toward lower Rab18 mRNA expression in obese T2D individuals compared to obese NG and IGT patients (specifically in omental fat in men), there does not appear to be an apparent association between the expression of this GTPase and insulin sensitivity.

In conclusion, our data provide novel insights concerning the distribution and function of Rab18 in adipocytes. Specifically, through its interaction with the surface of LDs and, most likely, with ER membranes, Rab18 regulates adipocyte lipid metabolism in response to both lipogenic (insulin) and lipolytic (β-adrenergic) inputs. In humans, Rab18 is associated with the surface of LDs in adipocytes and its expression in adipose tissue, which displays sex- and depot-related differences, correlates with increased adiposity, providing evidence for the participation of this GTPase in the regulation of human adipocyte biology under both normal and pathological conditions.

## Materials and Methods

### Cell culture and in vitro experimental setups

3T3-L1 cells [obtained from the American Type Culture Collection (Manassas, VA)] were differentiated into adipocytes, as previously described [60]. Briefly, 100% confluent cells (day 0) were incubated in DMEM containing 10% FBS, 0.5 mM isobutylmethylxanthine (IBMX), 0.25 µM dexamethasone and 10 µg/ml insulin for 72 h (day 3). The medium was replaced by DMEM with 10% FBS and 10 µg/ml insulin for an additional 72-h period (day 6) and was then exchanged by DMEM without insulin until day 10, when all the experiments were carried out.

Rab18 and adiponectin mRNA expression and protein content during adipocyte differentiation were assessed in 3T3-L1 cells at days 0, 3, 6 and 10 of differentiation. The hormonal control of Rab18 expression in 3T3-L1 cells was evaluated at day 10 of differentiation by preincubating cells in 1 ml serum-free culture medium for 2 h and then treating them with 10 µM isoproterenol, 100 nM dexamethasone, 10 nM GH, 4.8 nM PACAP38, 100 nM insulin, or 10 µM rosiglitazone. After a 24-h treatment period, cells were resuspended in 1 ml TRIzol Reagent (Invitrogen Corp., Barcelona, Spain) and processed for RNA analysis. After treatment of cells with 100 nM insulin or 10 µM isoproterenol for 24 h, Rab18 protein and adiponectin levels were determined by Western blot using an anti-rat Rab18 antibody (1∶500; Calbiochem, Barcelona, Spain) and an anti-mouse adiponectin antibody (1∶5000; Chemicon Int. CA).

The effect of insulin and isoproterenol on the intracellular distribution of Rab18 and the contribution of different components of the corresponding signaling pathways were investigated by immunocytochemistry. Specifically, 3T3-L1 cells were treated with insulin (100 nM) in the presence or absence of the PI3K inhibitor wortmannin (1 µM) for 4 h. Parallel cultures were exposed to isoproterenol (10 µM) alone or in combination with the AC blocker MDL 12,330A (1 µM) or with the PKA blocker H89 (1 µM) for 4 h. All inhibitors were added alone to cell cultures 90 min prior to the 4-h combined treatment.

### Quantification of Rab18 gene expression by real-time RT-PCR

For studies in 3T3-L1 cells, 2 µg of total RNA were used for RT and subsequent real-time PCR using SYBR Green tagging quantification in an iCycler IQ PCR detection system (Bio-Rad, Madrid, Spain). The specific primer pair used for Rab18 was 5′-CTCTGAAGATACTCATCATCGG-3′ sense and 5′-CCTCTCTTGACCAGCTGTATCCCA-3′ antisense, which amplify a 185-bp fragment, and that used for adiponectin was 5′-GTCCCGGAATGTTGCAGTAG-3′ sense and 5′-TGGAGAAGCCGCTTATGTGT-3′ antisense. As an endogenous reference gene, the 18S small subunit ribosomal RNA gene was amplified in parallel to Rab18 using the specific primer pair 5′-CCCATTCGAACGTCTGCCCTAT-3′ sense and 5′-TGCTGCCTTCCTTGGATGTGGTA-3′ antisense, which amplify a 137-bp fragment.

### Immunocytochemistry

3T3-L1 adipocytes were fixed with 4% paraformaldehyde (15 min), incubated with PBS containing 0.1% saponin and 1% BSA (1 h at RT), and exposed to anti-rat Rab18 antibody (1∶500; Calbiochem) or anti-human Rab18 (1∶100; Sigma Aldrich) in combination with guinea pig anti-human perilipin (1∶100; Progen, Heidelberg, Germany). When Rab18 was used in combination with mouse monoclonal anti-human calnexin (1∶50; Abcam, Cambridge, UK) or mouse monoclonal anti-rat protein disulfide isomerase (PDI) (1∶50; Abcam), cell permeabilization was assessed with PBS containing 0.3% Triton-X-100 and 1% BSA (1 h at RT). After incubation of cells with the primary antibodies overnight at 4°C, the immunoreaction was revealed with an anti-rabbit Alexa594-conjugated secondary antibody (1∶500; Invitrogen Corp.) alone or in combination with anti-guinea pig or anti-mouse Alexa488-conjugated secondary antibodies (1∶500; Invitrogen Corp.). Samples were visualized under a TCS-SP2-AOBS confocal laser scanning microscope (Leica Corp., Heidelberg, Germany). Image stacks were deconvoluted using the Huygens Essential software package (version 2.4.4; (SVI, Hilversum, The Netherlands). The degree of colocalization was estimated by determining an overlapping pixel map of the two fluorescent channels (*i.e.*, a mask) using the Colocalization Finder plugin for ImageJ 1.32 (NIH, Bethesda, MA), as previously shown [61] and Imaris 6.4 (Bitplane, Zurich, Switzerland). The colocalization was then quantified using Pearson's coefficient (PC), a value that is not sensitive to the intensity of the background or that of the overlapping pixels. This coefficient takes a value between −1 and +1, with −1 representing no overlap whatsoever between images and +1 a perfect overlap of the channels. Negative control samples without the primary antibody were included to assess non-specific staining.

### Western blot analysis

Protein extracts were obtained from cells lysed in buffer containing 50 mM Tris-HCl (pH 7.4), 150 mM NaCl, 1% Triton-X-100, 1 mM EDTA, and 1 µg/ml anti-protease cocktail, and then 150 µg of total protein was loaded onto 12.5% SDS-PAGE gels. Rabbit polyclonal antibodies against rat Rab18 (Calbiochem) and β-actin (Sigma-Aldrich, London, UK) were dispensed overnight at 4°C and peroxidase-conjugated secondary antibodies were administered for 1 h at room temperature. The immunoreaction was visualized using ECL plus (GE Healthcare, Buckinghamshire, UK) and band intensities were assessed using ImageJ 1.32 (NIH). Data were normalized to the corresponding β-actin band intensities.

### Subcellular fractionation

Subcellular fractionation was performed as previously described [62]. 3T3-L1 adipocytes were incubated in the presence or absence of 100 nM insulin and 10 µM isoproterenol for 4 h. Then, cells were rinsed with Ca^2+^- and Mg^2+^-free medium (D-PBS; Invitrogen Corp.) and resuspended in 3 ml lysis buffer containing 25 mM Tris-HCl, 100 mM KCl, 1 mM EDTA, 5 mM EGTA and 1 µg/ml anti-protease cocktail (pH 7.4). Cells were disrupted and mixed with an equal volume of lysis buffer containing 1.08 M sucrose and extracts were centrifuged at 1,500 *g* for 10 min. Supernatants were transferred to a 12-ml ultracentrifuge tube and sequentially overlaid with 2 ml each of 0.27 M and 0.135 M sucrose buffer and, finally, free-sucrose solution containing 25 mM Tris-HCl, 1 mM EDTA, and 1 mM EGTA (pH 7.4). After centrifugation at 130,000 *g* (1 h, 4°C), 8 fractions of 1.5 ml each were collected. After protein precipitation, extracts from each fraction were analyzed by Western blot as described above.

### Electroporation

3T3-L1 adipocytes were electroporated as previously described [63]. Briefly, cells cultured on 150-mm Petri dishes were mechanically resuspended, washed twice with D-PBS and submerged into 100 µl D-PBS containing 50 µg of the corresponding plasmids or 2 nmol siRNAs. Electroporation was performed in 2-mm thick electroporation cuvettes using a Gene Pulser Xcell (Bio-Rad), which delivered a 500 µF pulse at 110 V. After electroporation, cells were plated onto 35-mm dishes and allowed to recover in DMEM supplemented with 10% FBS for 48–72 h. For overexpression analysis, cells were electroporated with a phrGFP expression vector (mock-transfected cells), or with expression vectors coding for GFP-Rab18 or for a constitutively active mutant of Rab18 [namely, Rab18(Q67L)] fused to GFP, and cultured for 48 h prior to the experiments. For silencing studies, 2 nmols of a Rab18-targeted commercial double stranded siRNA oligonucleotide (Applied Biosystems-Ambion, Austin, TX) or a specific scramble negative control siRNA purchased from the same company were used. After electroporation, cells were kept in culture for 72 h before measuring lipolytic and lipogenic activities.

GFP-Rab18 overexpression was confirmed by fluorescence microscopy and Western blot, and Rab18 silencing rate was estimated by quantitative RT-PCR and Western blot.

### Analysis of LD number and size

3T3-L1 adipocytes were electroporated with either the GFP plasmid (*i.e.*, mock-transfected cells) or with the GFP-Rab18 overexpression vector and cultured for 48 h. After that, cells were maintained in serum-free medium for 2 h, and incubated in the presence or absence of 10 µM isoproterenol or 100 nM insulin for 1 h. Then, cells were fixed with 4% paraformaldehyde, washed with 60% isopropanol and incubated with Oil Red O solution (Sigma-Aldrich) for 30 min. Samples were then visualized by confocal microscopy. Ten cells per experimental condition were randomly selected and the average LD number and size per cell was estimated using ImageJ 1.32 (NIH).

### Assessment of lipogenesis and lipolysis

Lipogenesis was measured in 3T3-L1 cells either overexpressing GFP, GFP-Rab18 or GFP-Rab18(Q67L) vectors or silenced for Rab18 expression, treated or not with 100 nM insulin for 4 h. Cells were lysed in RIPA buffer (0.1% SDS, 1% Triton-X-100, 5 mM EDTA, 1 mM Tris HCl, 150 mM NaCl and 1% deoxycholate; pH 7.4) and intracellular triglycerides quantified using an Infinity™ Triglycerides Liquid Stable Reagent kit (Thermo Electron Corp., Grenoble, France). Triglyceride levels were normalized to total protein content.

Lipolysis was also quantified in overexpressing or silenced cells. To this end, cells were incubated in the presence or absence of 50 µM forskolin for 30 min and extracellular glycerol measured by using a Free Glycerol Reagent kit (Sigma-Aldrich). In this case, intracellular protein content was used for normalization.

### Human studies

Samples of omental and subcutaneous adipose tissue were obtained from the abdominal region of 46 Caucasian individuals (24 men and 22 women) undergoing either a Roux-en-Y gastric bypass (n = 34) or laparoscopic surgery procedures (Nissen fundoplication for hiatus hernia repair or cholecystectomies) (n = 12). Body mass index (BMI) was calculated as weight in kilograms divided by the square of height in meters and obesity was classified according to body mass index (BMI≥30 kg/m^2^. Obese patients were classified into three groups [normoglycemic (NG), with impaired glucose tolerance (IGT) or type 2 diabetes (T2D)] following the criteria of the Expert Committee on the Diagnosis and Classification of Diabetes, based on both fasting serum glucose concentrations and 2-h plasma glucose levels following an oral glucose tolerance test [64]. Subjects with T2D were not on insulin therapy or on medication likely to influence endogenous insulin levels. Immediately after removal, samples were washed in DMEM (Invitrogen Corp.) and divided into 2–3 pieces, which were either frozen in liquid nitrogen and stored at −80°C for subsequent gene expression analysis or immediately processed for the separation of mature adipocytes. All investigations reported were carried out in accordance with the principles of the Declaration of Helsinki as revised in 2008. The experimental design was approved by the Ethical Committees responsible for research of the Clínica Universidad de Navarra (Pamplona, Spain), Hospital Clínico Vírgen de la Victoria (Málaga, Spain) and Hospital Universitario Reina Sofía (Córdoba, Spain). Written informed consent was obtained from all participating patients.

All samples from the different patients were processed separately and considered as individual observations. RNA was extracted from each adipose sample using an RNeasy lipid tissue kit (Qiagen Madrid, Spain) following the manufacturer's protocol. Integrity and concentration of RNA were checked with a 2100 Bioanalyzer (Agilent Technologies, Santa Clara, CA). The expression levels of the Rab18 gene, and of 18S ribosomal RNA (rRNA) as housekeeping gene, were measured by RT-PCR using an iCyclerTM Real-Time PCR System (Bio-Rad Laboratories). Specifically, 1 µg of total RNA was reverse transcribed in a 20-µl final volume using random hexamers (Roche, Barcelona, Spain) as primers and 200 units of M-MLV reverse transcriptase (Invitrogen Corp.). Real-time PCR was carried out with 1 µl of cDNA and 24 µl of reaction mixture [12.5 µl of 2 X SYBR Green Supermix (Bio-Rad)], 9.5 µl of RNase-free water and 1 µl of the corresponding primers (Rab18: sense, 5′-CCCTGAAGATCCTCATCATCGG-3′; antisense, 5′-CCTCTCTTGACCAGCAGTATCCCA-3′ and 18S: sense, 5′-CCCATTCGAACGTCTGCCCTATC-3′; antisense, 5′-TGCTGCCTTCCTTGGATGTGGTA-3′). After an initial hold of 2 min at 94°C, samples were cycled 40 times at 94°C for 15 s and at 61°C for 15 s. For quantitative analysis, a standard curve-based method was used for relative real-time PCR data processing. The expression of Rab18 gene was normalized to that of the housekeeping gene. All measurements were performed in duplicate and the average values were calculated. Controls consisting of reaction mixture without cDNA were negative in all runs.

An additional set of adipose tissue samples from lean and obese women and men was employed for assessment of Rab18 protein levels. To this end, tissues were homogenized and levels of Rab18 protein were assessed by immunoblotting as described earlier for 3T3-L1 cells.

For the isolation of mature adipocytes, freshly isolated subcutaneous and omental samples were incubated in Krebs-Ringer Hepes medium (119 mM NaCl, 4.7 mM KCl, 1.2 mM MgSO_4_, 2.5 mM CaCl_2_, 1.2 mM KH_2_PO_4_, 20 mM Hepes pH 7.4, 2 mM glucose, 2% BSA) with 400 a.u./ml of collagenase (Sigma-Aldrich) at 37°C for 1 h in a shaking bath. Undigested tissue was removed by filtering through a sterile 100 µm pore Cell Strainer (BD Falcon, CA) and centrifuged at 600 *g* for 10 min to separate the floating mature adipocyte layer. Mature adipocytes were washed with DMEM and then processed for Rab18 immunostaining as described above.

### Statistical analysis

Data from 3T3-L1 cells were obtained from a minimum of three replicate wells per treatment from, at least, three independent experiments. Average LD size was assessed from 200–300 LDs from 10 randomly selected cells per experimental condition. Single comparisons were performed using the Student's *t* test, while multiple comparisons were assessed by one-way ANOVA followed by a Newman-Keuls test using GraphPad Prism 4 (La Jolla, CA). Differences were considered statistically significant if *P*<0.05.

## Supporting Information

Figure S1 Associated with Figure 6. Effect of Rab18 overexpression on LD size. (A) GFP (top panels) or GFP-Rab18 (bottom panels) transfected 3T3-L1 adipocytes were incubated in the absence (left panels) or presence of 100 nM insulin (middle panels) or 10 µM isoproterenol (right panels) for 1 h. LDs were visualized by Oil-Red-O staining as indicated in “Material and Methods”. GFP was always found diffuse within cells irrespective of the treatment, whereas GFP-Rab18 accumulated, at different extent, around LDs that exhibit an ample range of sizes. Scale bars, 5 µm. (B) High magnification 3D reconstruction of Oil-Red-O-stained LDs within a cell expressing GFP-Rab18. Twenty confocal slices of a 3T3-L1 adipocyte were projected in a single image and a region of interest renderized using Imaris 6.4. software (Bitplane, Zurich, Switzerland). As shown, exogenous Rab18 adopts a ring-like distribution around LDs, similarly to that observed for endogenous Rab18. Scale bar, 0.25 µm. (C) Analysis of the average LD size revealed that Rab18 overexpression induces a general increase in LD size irrespective of the treatment. Data are presented as the mean ± SEM of, at least, 200 LDs per experimental group. *, *P*<0.05, **, *P*<0.01, and ***, *P*<0.001 *vs.* groups indicated in the graph.
(TIF)

Figure S2 Associated with Figure 8. Rab18 localization in dispersed, mature human adipocytes. Representative confocal images of human adipocytes from omental (top panels) and subcutaneous (bottom panels) fat depots. Adipose tissue samples were enzymatically and mechanically dispersed as indicated in “Material and Methods”. Then, cells were processed for immunostaining against Rab18. A single middle plane is shown. Rab18 immunoreactivity was found around the large LD characteristic of mature adipocytes, as well as around small LDs (insets). DAPI was used for labeling nuclei. Scale bars; 20 µm.
(TIF)

Table S1 Associated with Figure 8. Rab18 cDNA copy number/18S cDNA copy number in omental and subcutaneous adipose tissue from lean and obese women and men, as determined by quantitative RT-PCR. Values represent means ± SEM of, at least, 4 individuals, a, *P*<0,05 *vs.* omental adipose tissue from lean men; b, *P*<0,05 *vs.* omental adipose tissue from lean women; c, *P*<0,05 *vs.* omental adipose tissue from lean men; d, *P*<0,001 *vs.* omental adipose tissue from obese men.
(DOCX)
