# Supplementary figures and images for: Rab18 Dynamics in Adipocytes in Relation to Lipogenesis, Lipolysis and Obesity
Source: PLoS One. 2011 Jul 28;6(7):e22931. doi: 10.1371/journal.pone.0022931 (PMC3145781; doi:10.1371/journal.pone.0022931)

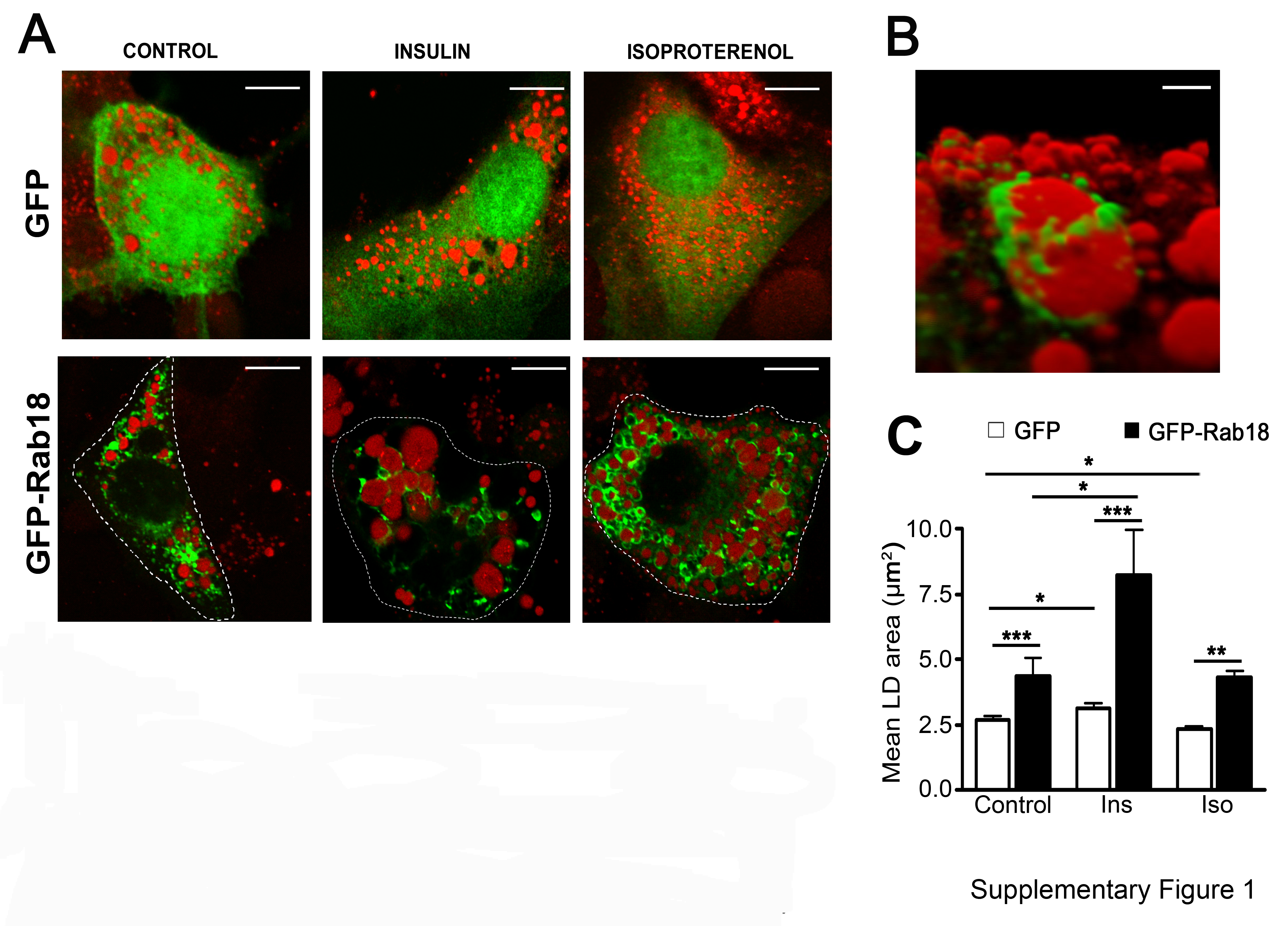

Supplement: Figure S1 — Associated with Figure 6. Effect of Rab18 overexpression on LD size. (A) GFP (top panels) or GFP-Rab18 (bottom panels) transfected 3T3-L1 adipocytes were incubated in the absence (left panels) or presence of 100 nM insulin (middle panels) or 10 µM isoproterenol (right panels) for 1 h. LDs were visualized by Oil-Red-O staining as indicated in “Material and Methods”. GFP was always found diffuse within cells irrespective of the treatment, whereas GFP-Rab18 accumulated, at different extent, around LDs that exhibit an ample range of sizes. Scale bars, 5 µm. (B) High magnification 3D reconstruction of Oil-Red-O-stained LDs within a cell expressing GFP-Rab18. Twenty confocal slices of a 3T3-L1 adipocyte were projected in a single image and a region of interest renderized using Imaris 6.4. software (Bitplane, Zurich, Switzerland). As shown, exogenous Rab18 adopts a ring-like distribution around LDs, similarly to that observed for endogenous Rab18. Scale bar, 0.25 µm. (C) Analysis of the average LD size revealed that Rab18 overexpression induces a general increase in LD size irrespective of the treatment. Data are presented as the mean ± SEM of, at least, 200 LDs per experimental group. *, P<0.05, **, P<0.01, and ***, P<0.001 vs. groups indicated in the graph. (TIF) [file pone.0022931.s001.tif]

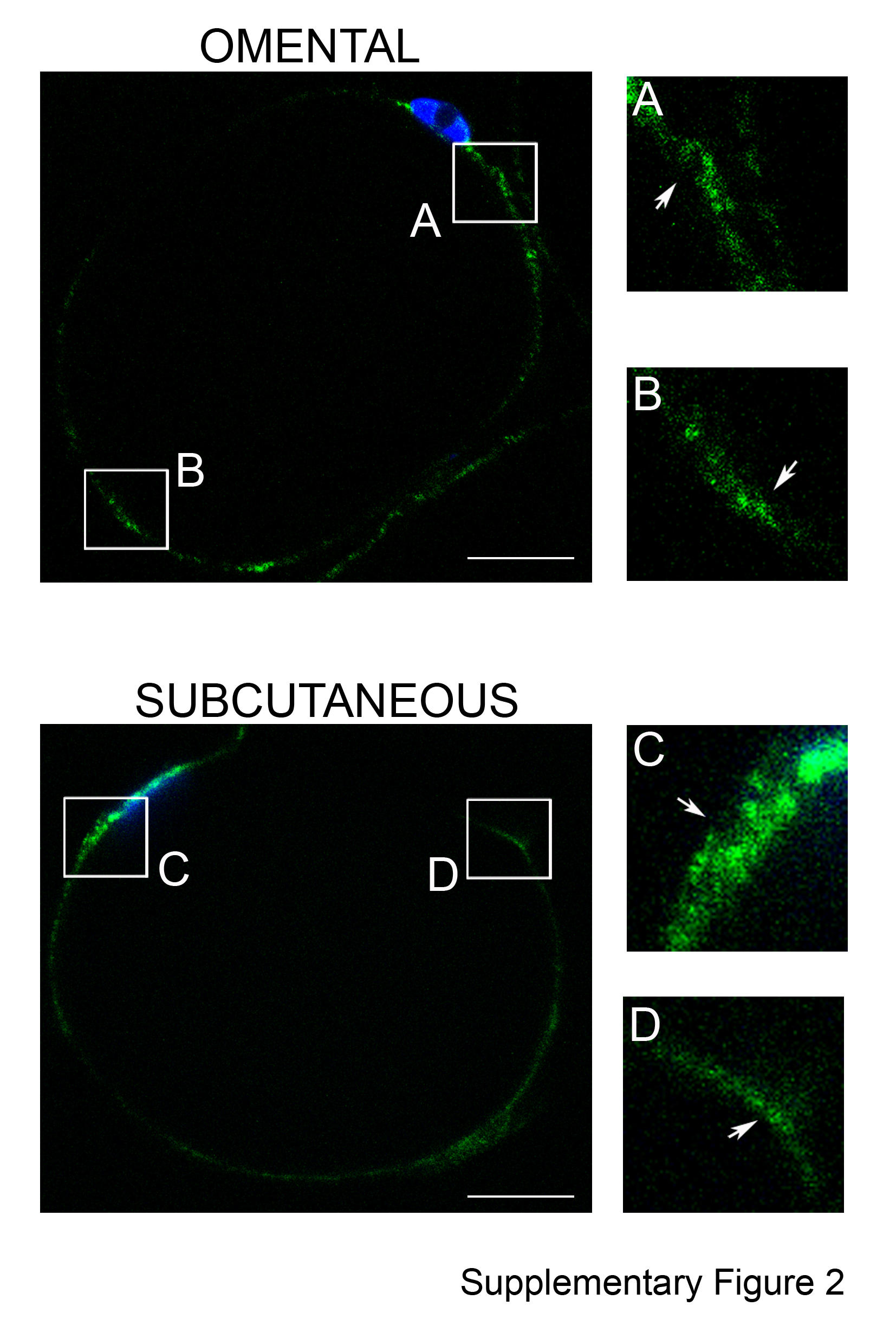

Supplement: Figure S2 — Associated with Figure 8. Rab18 localization in dispersed, mature human adipocytes. Representative confocal images of human adipocytes from omental (top panels) and subcutaneous (bottom panels) fat depots. Adipose tissue samples were enzymatically and mechanically dispersed as indicated in “Material and Methods”. Then, cells were processed for immunostaining against Rab18. A single middle plane is shown. Rab18 immunoreactivity was found around the large LD characteristic of mature adipocytes, as well as around small LDs (insets). DAPI was used for labeling nuclei. Scale bars; 20 µm. (TIF) [file pone.0022931.s002.tif]
