# Supplementary material for: Rab18 Dynamics in Adipocytes in Relation to Lipogenesis, Lipolysis and Obesity
Source: PLoS One. 2011 Jul 28;6(7):e22931. doi: 10.1371/journal.pone.0022931 (PMC3145781; doi:10.1371/journal.pone.0022931)
Supplement: Table S1 — Associated with Figure 8. Rab18 cDNA copy number/18S cDNA copy number in omental and subcutaneous adipose tissue from lean and obese women and men, as determined by quantitative RT-PCR. Values represent means ± SEM of, at least, 4 individuals, a, P<0,05 vs. omental adipose tissue from lean men; b, P<0,05 vs. omental adipose tissue from lean women; c, P<0,05 vs. omental adipose tissue from lean men; d, P<0,001 vs. omental adipose tissue from obese men. (DOCX) [file pone.0022931.s003.docx]

|  |  | **Omental Adipose Tissue** | | **Subcutaneous Adipose Tissue** | |
| --- | --- | --- | --- | --- | --- |
|  |  | **Mean** | **SEM** | **Mean** | **SEM** |
| **Lean** | **Women** | 3.79x10^-6 a^ | 7.47x10^-7^ | 3.16x10^-5 b^ | 1.38x10^-5^ |
|  | **Men** | 1.127x10^-6^ | 4.49x10^-7^ | 1.42x10^-5 c^ | 6.35x10^-6^ |
| **Obese** | **Women** | 1.10x10^-5^ | 9.98x10^-6^ | 4,13x10^-5^ | 4.37x10^-5^ |
|  | **Men** | 9.41x10^-5^ | 2.05x10^-6^ | 4.13x10^-5 d^ | 3.85x10^-6^ |

**Table S1**
